# Quality of life in children and adolescents with bipolar I depression treated with olanzapine/fluoxetine combination

**DOI:** 10.1186/s13034-017-0170-7

**Published:** 2017-07-12

**Authors:** Daniel J. Walker, Melissa P. DelBello, John Landry, Deborah N. D’Souza, Holland C. Detke

**Affiliations:** 1 Eli Lilly and Company, Lilly Research Laboratories, Lilly Corporate Center, Indianapolis, IN 46285 USA; 2 Division of Bipolar Disorders Research, Department of Psychiatry and Behavioral Neuroscience, University of Cincinnati College of Medicine, Cincinnati, OH USA; 3 Eli Lilly Canada Inc., Toronto, Canada; 4 inVentiv Health Company, LLC, Indianapolis, IN USA

**Keywords:** Olanzapine fluoxetine combination, Bipolar, Depression, Quality of life, Children, Adolescents

## Abstract

**Background:**

We examined the efficacy of olanzapine/fluoxetine combination (OFC) in improving health-related quality of life (QoL) in the treatment of bipolar depression in children and adolescents.

**Methods:**

Patients aged 10–17 years with bipolar I disorder, depressed episode, baseline children’s depression rating scale-revised (CDRS-R) total score ≥40, Young Mania Rating Scale (YMRS) total score ≤15, and YMRS-item 1 ≤ 2 were randomized to OFC (6/25–12/50 mg/day olanzapine/fluoxetine; n = 170) or placebo (n = 85) for up to 8 weeks of double-blind treatment. Patients and parents completed the revised KINDL questionnaire for measuring health-related QoL in children and adolescents (KINDL-R) at baseline and endpoint. The mean change in CDRS-R total and item scores were used to compare improvement in symptomatology in patients taking OFC and placebo. Tests were 2-sided using a Type I error cutoff of 0.05, and no adjustments for multiple comparisons were made.

**Results:**

Baseline QoL as measured by the KINDL-R was substantially impaired relative to published norms for a healthy school-based sample. OFC-treated patients demonstrated an improvement over placebo at endpoint with respect to mean change from baseline in the patient-rated KINDL-R Self-esteem subscale score (*p* = 0.028), and in the parent KINDL-R ratings of emotional well-being (*p* = 0.020), Self-esteem (*p* = 0.030), and Family (*p* = 0.006). At endpoint, OFC-treated patients still had a lower QoL compared to the normative population. OFC showed significant improvement (*p* ≤ 0.05) versus placebo on the CDRS-R total score and on 7 of the 17 CDRS-R items.

**Conclusions:**

Patients aged 10–17 years with an acute episode of bipolar depression and their parents reported greater improvements (parents noticed improvements in more areas than did their offspring) on some aspects of QoL when treated with OFC compared with placebo. However, after 8 weeks of treatment, KINDL-R endpoint scores remained lower than those of the, presumably healthy, control population.

*Clinical trial registration information* A Study for Assessing Treatment of Patients Ages 10–17 with Bipolar Depression; http://www.clinicaltrials.gov; NCT00844857

## Background

The number of children and adolescents diagnosed with bipolar disorder has increased in recent years [1, 2]. A meta-analysis of 12 international epidemiological studies reported that the overall occurrence of pediatric bipolar disorder was 1.8% [3], and in an epidemiological sample of more than 10,000 adolescents, the prevalence of bipolar disorder was reported to be 2.9% [4].

There is a significant reduction in quality of life (QoL) in youth with bipolar disorder [5–7], with patients experiencing social and attention problems, delinquent and aggressive behavior, and a poor ability to maintain stable relationships and perform successfully at work or school [8–12]. Improving the QoL of patients with bipolar disorder is an important aspect of a successful treatment outcome. Although studies have examined the effect of treatment of mania on QoL in bipolar disorder [13, 14], health-related QoL in bipolar depression has not been studied extensively.

A recent 8-week, double-blind, placebo-controlled study demonstrated that the combination of olanzapine and fluoxetine (OFC) was significantly more efficacious than placebo for the acute treatment of bipolar depression in children and adolescents (aged 10–17 years) [15]. In this study [15], the mean improvement in children’s depression rating scale-revised (CDRS-R) [16] total score was significantly greater for OFC-treated patients than for placebo-treated patients starting at week 1 and for all subsequent visits up to week 8, and the rates of and times to response and remission were also significantly greater for OFC- than placebo-treated patients. The most common treatment-emergent adverse events in the OFC group in this study were somnolence, weight gain, and increased appetite. Based on this study, OFC was the first medication approved by the US Food and Drug Administration (USFDA) for the treatment of depressive episodes associated with bipolar I disorder in children and adolescents [17].

Antipsychotic monotherapy treatment has been shown to be effective for treating children and adolescents with bipolar disorder [18, 19] including improvement in manic symptoms in youth with bipolar disorder. However, corresponding improvements in QoL have been mixed. For example, in a 4-week study of manic youth (aged 10–17 years) with bipolar disorder, there were no significant differences between aripiprazole and placebo (at week 4) in QoL as measured by the change in total score on the Pediatric QoL Enjoyment and Satisfaction Questionnaire [20]. However, another study of bipolar adolescents (mean age 15 years) with manic or mixed episodes reported that significant improvements in health-related QoL (child health questionnaire), especially in psychosocial domains, were observed after 28 days of treatment with quetiapine [21]. These improvements were not significantly related either to changes in mania or depressive symptoms.

Olanzapine monotherapy is approved by the USFDA for the treatment of schizophrenia and manic or mixed episodes of bipolar I disorder in adolescents, based on a study of patients with schizophrenia [22] and on a study in patients with bipolar I mania [23]. Treatment with olanzapine was also effective on multiple domains of psychosocial functioning compared with placebo when examining health-related QoL in manic adolescents with bipolar disorder [24]. Compared with the placebo group, patients in the olanzapine group showed significantly greater improvement in the psychosocial summary score from baseline to endpoint and in the mean change scores of behavior, family activities, and mental health subscales of the child health questionnaire-parental form 50 [24].

The selective serotonin reuptake inhibitor (SSRI) fluoxetine is an antidepressant that is approved in children and adolescents for the treatment of major depressive disorder [25, 26] and for the treatment of obsessive–compulsive disorder [27]. Fluoxetine has been shown to improve global health, functioning, and QoL in depressed adolescents [28]. However, there is a paucity of studies assessing changes in QoL in children or adolescents with bipolar depression treated with either an SSRI or an antipsychotic.

This paper focuses on data from a previously published three-country, multi-site, double-blind, placebo-controlled trial [15] demonstrating the efficacy of OFC compared with placebo during 8 weeks of treatment of depressive episodes associated with bipolar I disorder in children and adolescents. The present analysis evaluates mental health outcomes from that study to determine whether OFC was superior to placebo in improving QoL.

## Methods

### Patients

Patients who were aged 10–17 years and met *Diagnostic and Statistical Manual of Mental Disorders, Fourth Edition, Text Revision* (DSM-IV-TR) [29] diagnostic criteria for bipolar I disorder, current episode depressed, as confirmed by the Kiddie-Schedule for Affective Disorders-Present and Lifetime [30], were recruited for study participation [15]. To be included in the trial, patients were required to have a CDRS-R total score ≥40 and a Young Mania Rating Scale (YMRS) [31] total score ≤15, with a YMRS item 1 (elevated mood) score of ≤2. Patients were excluded from study participation if they were, in the opinion of the investigator, actively suicidal; had an acute, serious or unstable medical condition; had clinically significant laboratory abnormalities; had one or more seizures of unclear etiology; or had a current or lifetime diagnosis (DSM-IV-TR criteria) of schizophrenia, schizophreniform disorder, schizoaffective disorder, delusional disorder, psychotic disorder, delirium, amnestic disorder, any substance-induced disorder, mental retardation, substance dependence other than nicotine or caffeine within 30 days prior to study entry, or a current diagnosis of autism or pervasive developmental disorder.

### Study design and treatment

This study was conducted across 41 sites (29 in the United States, 4 in Mexico, and 8 in Russia). Patients were randomized in a 2:1 ratio to treatment with OFC or placebo for up to 8 weeks of double-blind treatment. Patients in the OFC group were initiated at a dose of 3/25 mg (olanzapine/fluoxetine doses), which was increased to 6/25 mg at day 3, 6/50 mg at week 1, and 12/50 mg at week 2, with flexible dosing thereafter among the allowed doses of 6/25, 6/50, 12/25, and 12/50 mg. Limited adjunctive use of benzodiazepines was permitted, and anticholinergic therapy was permitted for treatment of extrapyramidal symptoms.

The study protocol was approved by the ethical review board at each study center and was conducted in full accordance with ethical principles of good clinical practice (GCP) and the Declaration of Helsinki and its guidelines. Informed assent and consent were obtained from all patients and their legal guardian, respectively, at study entry and before commencement of any study procedures.

### Health outcome measures

Patients’ QoL was rated independently by patients and their parent or guardian using the revised Kinder Lebensqualitatsfragebogen-überarbeitet or KINDL questionnaire for measuring health-related QoL in children and adolescents (KINDL-R) [32]. The KINDL-R is a 31-item questionnaire for measuring health-related QoL in children and adolescents [33], revised by Ravens-Sieberer and Bullinger [34]. It consists of 24 Likert-scaled items grouped into 6 subscales measuring specific aspects of QoL (physical well-being, emotional well-being, self-esteem, family, friends, and school) consisting of 4 items each, followed by an additional module entitled “Disease.” The Disease module assesses perceptions of how the illness itself impacts the patient (e.g., the patient’s feelings regarding the disease, ability to cope with the disease, and sense of being treated differently by others because of the disease) and is limited to those patients self-identifying as hospitalized or having a long-term illness. Subscale scores are produced by combining the item ratings for each of the 6 subscales and converting each subscale score to a scale of 0–100, with higher scores representing better QoL. Similarly, a total score is produced by combining the item ratings across all 6 subscales (not including the Disease module) and converting this score to a scale of 0–100.

The KINDL-R is a validated scale [34] that is used as a health outcome measure across a range of health and mental health issues. The KINDL-R scale has been translated into numerous languages including Spanish and Russian. The determination of the reliability and validity of the KINDL-R questionnaire can be located in the manual [34]. The internal consistency (Cronbach’s alpha) of the overall scale was found to be 0.84 and 0.89 in the child and parent versions, respectively. Ratings were conducted at the time of randomization (baseline) and at the patient’s last study visit (endpoint). Scores are interpreted relative to normative data provided by the scale’s authors [32], which include those of a German adolescent student population (n = 583) with a mean age of 14.1 years.

### Measure of depressive symptomatology

The primary measure of efficacy was the mean change in CDRS-R [16] total score from baseline to week 8. The CDRS-R is a clinical interview tool and rating scale (patients are rated on 17 items), designed to assess the presence and severity of depression in children aged 6–12 years; it has also been used successfully for adolescents [35, 36]. The CDRS-R has been shown to be a reliable and valid measure in adolescents [37]. The CDRS-R was conducted at all study visits by trained raters. Item-level results for the CDRS-R items are included in the present analysis as these could potentially elucidate any findings with respect to QoL. Several of the CDRS-R items such as low self-esteem and impaired schoolwork are similar to subscales in the KINDL-R. It was of interest to determine if these items were similarly improved in a physician-based interview in tandem with self-reports by parents and children/adolescents.

### Statistical analyses

All analyses on KINDL-R patient- and parent-rated scales were based on the standardized total and standardized subscale scores and required both a baseline and post-baseline assessment (at endpoint visit) for inclusion in the analysis. Health outcomes analyses included comparisons of treatment groups with regard to mean change from baseline to endpoint in the total score and subscale scores separately for the patient- and parent-rated KINDL-R. Differences in KINDL-R scores between treatment groups were assessed using an analysis of covariance (ANCOVA) which included the covariates of baseline score and country in the model. All tests were 2-sided using a Type I error of 0.05 with no adjustments for multiple comparisons, so the results only provide inductive evidence.

For missing data, the ANCOVA models use last observation carried forward, and the mixed-model repeated measures (MMRM) models use restricted likelihood estimates under the missing at random assumption. Within-group changes were analyzed by t tests of least squares (LS) mean change (baseline to endpoint) within the treatment group. Mean change analyses for the CDRS-R scale used MMRM methodology as outlined in the primary manuscript [15] and report LS means from the model.

Due to the conceptual overlap between some items of the KINDL-R and the CDRS, a post hoc analysis was also conducted in which differences in KINDL-R scores between groups were assessed as above but with CDRS-R total score (change from baseline) also included as a covariate in the model to examine potential effects of the covariance between the two measures on treatment group differences.

A post hoc calculation of effect size was conducted for the treatment difference in KINDL scores using partial eta squared statistics. For the eta squared statistic a value of 0.02 is small, 0.13 is medium and 0.26 is large [38].

## Results

### Patient demographics and baseline characteristics

A total of 370 patients were screened, with 291 randomly assigned to OFC or placebo. Of these randomized patients, 36 were excluded from analysis for either not receiving a verifiable dose of study drug prior to discontinuing the study (n = 4) or for GCP violations at the study site (n = 32), leaving a total of 255 patients with evaluable data. A total of 221 (87%) of 255 patients and 218 (85%) parents/guardians had data available for inclusion in the KINDL-R analyses. Twelve of the patients were inpatients, and the rest were outpatients. A total of 176 (69.0%) patients completed the study, with no significant differences between treatment groups regarding study completion rate or reasons for discontinuation. The most common adverse event leading to discontinuation in the OFC group was weight increased (2.9%).

There were 188 patients from the United States (OFC, n = 123; placebo, n = 65), 44 from Russia (OFC, n = 31; placebo, n = 13), and 23 from Mexico (OFC, n = 16; placebo, n = 7). Baseline characteristics are presented in Table 1 and were comparable between the two treatment groups. There were 39 (15%) children younger than age 12 years (29 in the OFC group; 10 in the placebo group). Other baseline physical characteristics, including mean weight, height, and body mass index, were similar across treatment groups. Baseline ratings were consistent with an acutely ill patient population, with depression of moderate severity in the current episode. In all, 61% of patients reported that they had received at least 1 psychiatric medication in the past year, with the most frequently reported medications being risperidone (14%), quetiapine (12%), aripiprazole (9%), and valproic acid (9%).

**Table 1:** Baseline patient demographics and clinical characteristics

Variable	OFC (N = 170)	Placebo (N = 85)	Total (N = 255)
Age (years), mean (SD)	14.6 (2.3)	15.0 (2.1)	14.7 (2.3)
Sex (male), n (%)	84 (49.4)	46 (54.1)	130 (51.0)
Race (white), n (%)	119 (70.0)	61 (71.8)	180 (70.6)
Ethnicity (hispanic), n (%)	38 (22.4)	23 (27.1)	61 (23.9)
BMI, kg/m^2^, mean (SD)	23.5 (5.54)	24.0 (5.81)	23.7 (5.62)
Number of previous episodes of depression, median (range)	2.0 (0.0–80.0)	2.0 (0.0–50.0)	2.0 (0.0–80.0)
CDRS-R total score, mean (SD)	54.6 (10.0)	53.7 (8.1)	54.3 (9.4)
YMRS total score, mean (SD)	6.1 (3.8)	6.2 (3.9)	6.1 (3.8)
CGI-BP overall severity, mean (SD)	4.4 (0.7)	4.3 (0.7)	4.4 (0.7)

*BMI* body mass index, *CDRS-R* children’s depression rating scale-revised, *CGI-BP* clinical global impressions-bipolar, *N* number of patients, *OFC* olanzapine/fluoxetine combination, *SD* standard deviation, *YMRS* Young Mania Rating Scale

**Fig. 1:**
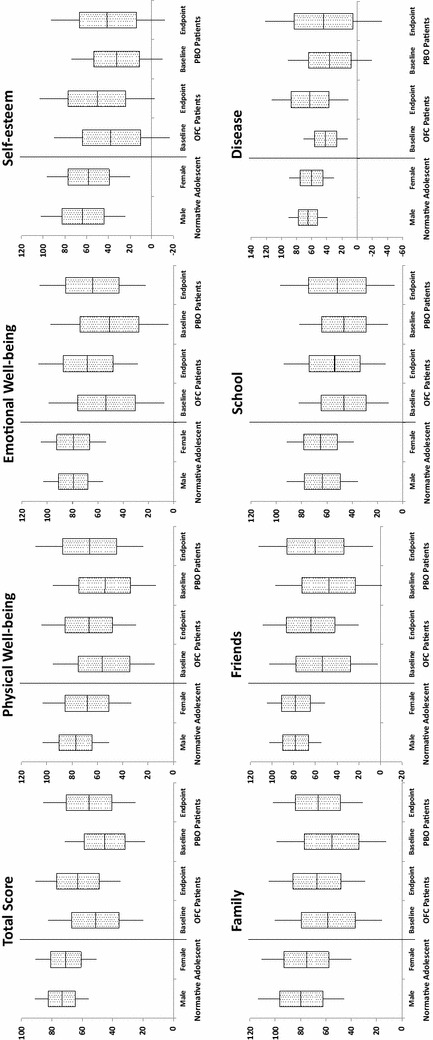
Changes in KINDL-R patient scale from baseline to endpoint relative to the normative data for adolescent males and adolescent females [32]. The* upper and lower edge of each box* represents the ‘mean plus one standard deviation’, and the ‘mean minus one standard deviation’ respectively, the *lines above and below each box* represent the ‘mean plus two standard deviations’, and the ‘mean minus two standard deviations’ respectively. The *line across the middle of each box* represents the mean. *KINDL-R* KINDL questionnaire for measuring health-related quality of life in children and adolescents, *OFC* olanzapine/fluoxetine combination, *PBO* placebo

The KINDL-R patient-rated and KINDL-R parent-rated scale baseline total scores for patients were 51.2 and 46.7, respectively, in the OFC group and 45.2 and 47.1, respectively, in the placebo group (Tables 2, 3). Figure 1 presents the results for OFC-treated patients from the KINDL-R patient-rated baseline and endpoint relative to the normative data for healthy children [32].

**Table 2:** Changes in KINDL-R patient-rated scale from baseline to endpoint (last observation carried forward)

KINDL-R domains	OFC			Placebo			Between-group *p* value^a^
	N	Mean baseline (SD)	LS mean change (SE)	N	Mean baseline (SD)	LS mean change (SE)	
Total	107	51.2 (15.6)	12.8 (1.7)	40	45.2 (13.2)	7.9 (2.4)	0.050
Physical well-being	151	54.9 (20.1)	12.8 (2.2)	70	54.4 (20.4)	12.4 (2.8)	0.873
Emotional well-being	150	53.3 (22.9)	13.7 (2.1)	70	51.0 (23.3)	11.1 (2.7)	0.330
Self-esteem	150	36.9 (26.7)	18.2 (2.7)	69	32.3 (20.9)	10.7 (3.4)	0.028
Family	147	58.2 (21.1)	9.6 (2.0)	68	55.5 (21.6)	9.6 (2.6)	0.994
Friends	147	52.8 (25.0)	14.8 (2.5)	62	47.9 (24.6)	12.2 (3.2)	0.427
School	110	46.9 (17.8)	6.5 (2.3)	44	46.6 (17.5)	4.6 (3.3)	0.559
Disease	10	41.7 (14.6)	12.3 (7.2)	3	36.1 (27.7)	−9.8 (13.6)	0.152

*KINDL-R* KINDL questionnaire for measuring health-related quality of life in children and adolescents, *LS* least squares, *N* number of patients, *OFC* olanzapine/fluoxetine combination, *SD* standard deviation, *SE* standard error

^a^Difference between LS mean change scores

**Table 3:** Changes in KINDL-R parent-rated scale from baseline to endpoint (last observation carried forward)

KINDL-R domains	OFC			Placebo			Between-group *p* value^a^
	N	Mean baseline (SD)	LS mean change (SE)	N	Mean baseline (SD)	LS mean change (SE)	
Total	83	46.7 (12.4)	16.0 (1.9)	39	47.1 (11.5)	10.9 (2.6)	0.066
Physical well-being	146	52.8 (20.0)	13.8 (2.3)	72	55.5 (22.0)	11.7 (2.9)	0.475
Emotional well-being	140	49.2 (20.1)	22.6 (2.3)	71	47.1 (16.7)	15.8 (2.9)	0.020
Self-esteem	144	33.3 (22.1)	20.3 (2.4)	69	29.9 (20.5)	13.6 (3.0)	0.030
Family	146	48.0 (20.5)	18.4 (2.1)	71	55.0 (19.5)	11.0 (2.7)	0.006
Friends	139	51.1 (19.9)	18.2 (2.3)	62	53.9 (20.4)	13.7 (3.0)	0.137
School	94	45.7 (15.0)	9.0 (2.0)	43	49.9 (14.6)	3.7 (2.7)	0.057
Disease	8	53.1 (25.1)	11.0 (7.4)	6	47.2 (30.2)	5.3 (7.9)	0.603

*KINDL-R* KINDL questionnaire for measuring health-related quality of life in children and adolescents, *LS* least squares, *N* number of patients, *OFC* olanzapine/fluoxetine combination, *SD* standard deviation, *SE* standard error

^a^Difference between LS mean change scores

### Dosing

The median daily doses of olanzapine and fluoxetine during the study were 7.8 mg (minimum–maximum: 3.0–10.6) and 40.8 mg (minimum–maximum: 20.8–47.7), respectively.

### Quality of life

Based on patients’ self-ratings, patients treated with OFC showed significant within-group improvement at a level of *p* < 0.001 for the total and subscale scores except for School at *p* = 0.006 and Disease (nonsignificant, *p* = 0.128). The placebo group also showed significant within-group improvement at a level of *p* < 0.001 for 4 subscale scores and was significant for the Total score (*p* = 0.001) and self-esteem subscale (*p* = 0.002) but was nonsignificant on School (*p* = 0.162) and Disease (*p* = 0.496). Based on patients’ self-ratings, patients treated with OFC demonstrated an improvement over placebo at endpoint with respect to mean change from baseline in the KINDL-R subscale score of Self-esteem (mean change 18.2 vs 10.7; *p* = 0.028) (Table 2). The mean change from baseline for the other KINDL-R patient-rated subscales and the Disease module were not significantly different between OFC-treated and placebo-treated patients. The effect sizes for the total and each of the subscales were small (less than 0.03) including 0.0224 for Self-esteem. The effect size was large for the Disease module (0.2696) but was based on just 13 patients.

Based on parents’ ratings, patients treated with OFC showed significant within-group improvement (*p* < 0.001) for the total and subscale scores except for Disease (*p* = 0.182). In addition, patients treated with OFC demonstrated an improvement over placebo at endpoint with respect to mean change from baseline in the KINDL-R parent-rated subscale scores of emotional well-being (mean change 22.6 vs 15.8; *p* = 0.020), Self-esteem (mean change 20.3 vs 13.6; *p* = 0.030), and Family (mean change 18.4 vs 11.0; *p* = 0.006) (Table 3). However, the mean change in the KINDL-R parent-rated scale total score for OFC (16.0) versus placebo (10.9) was not statistically significant (*p* = 0.066). The mean change from baseline for the other KINDL-R parent-rated subscales and the Disease module were not significantly different between the two treatment groups. The effect sizes were small (less than 0.04) for the total and subscale scores including emotional well-being (0.0261), Self-esteem (0.0226) and Family (0.0351). The effect size was also small for the Disease module (0.0312).

When the CDRS-R total score was added to the ANCOVA model, none of the KINDL-R patient-rated or KINDL-R parent-rated total and subscale scores were significantly different between the OFC and placebo treatment groups, indicating a strong degree of overlap in variance between the 2 measures.

Changes in the CDRS-R item scores from baseline to week 8 are shown in Table 4. The mean change from baseline was significantly in favor of OFC compared with placebo on the CDRS-R total score (−28.4 vs −23.4; *p* = 0.003). Seven of the items were also significantly improved with OFC, including 4 items (impaired schoolwork, difficulty having fun, social withdrawal, and low self-esteem) that assess a similar domain as some of the KINDL-R subscales.

**Table 4:** Changes in CDRS-R Item scores from baseline to week 8 (mixed-model repeated measures)

CDRS-R item (Item #)	OFC, N = 170 mean baseline (SD)	OFC, N = 170 LS mean change (SE)	Placebo, N = 84 mean baseline (SD)	Placebo, N = 84 LS mean change (SE)	Between-group *p* value
Total	54.6 (10.0)	−28.4 (1.1)	53.7 (8.2)	−23.4 (1.5)	0.003
Impaired schoolwork (1)	4.1 (1.4)	−1.92 (0.1)	4.0 (1.3)	−1.5 (0.2)	0.019
Difficulty having fun (2)	4.5 (1.2)	−2.7 (0.1)	4.7 (1.1)	−2.2 (0.2)	0.023
Social withdrawal (3)	3.9 (1.2)	−2.3 (0.1)	4.0 (1.2)	−1.8 (0.2)	0.003
Sleep disturbance (4)	3.4 (1.3)	−2.1 (0.1)	3.4 (1.3)	−1.3 (0.1)	<.001
Appetite disturbance (5)	2.4 (1.2)	−0.5 (0.1)	2.7 (1.3)	−0.8 (0.2)	0.171
Excessive fatigue (6)	4.0 (1.5)	−2.3 (0.1)	3.9 (1.7)	−2.2 (0.2)	0.488
Physical complaints (7)	2.8 (1.5)	−1.4 (0.1)	2.8 (1.4)	−1.2 (0.1)	0.170
Irritability (8)	4.3 (1.5)	−2.3 (0.1)	4.1 (1.5)	−1.7 (0.2)	0.004
Excessive guilt (9)	2.7 (1.5)	1.5 (0.1)	2.5 (1.5)	−1.2 (0.1)	0.053
Low self-esteem (10)	3.8 (1.4)	−2.1 (0.1)	3.6 (1.3)	−1.7 (0.2)	0.019
Depressed feelings (11)	4.6 (1.1)	−2.9 (0.1)	4.5 (1.1)	−2.4 (0.2)	0.005
Morbid ideation (12)	2.2 (1.3)	−0.8 (0.1)	2.0 (1.3)	−0.8 (0.1)	0.780
Suicidal ideation (13)	1.3 (0.7)	−0.2 (0.1)	1.2 (0.6)	−0.16 (0.1)	0.430
Excessive weeping (14)	2.8 (1.6)	−1.5 (0.1)	2.5 (1.6)	−1.5 (0.1)	0.897
Depressed facial affect (15)	3.4 (1.2)	−1.9 (0.1)	3.2 (1.2)	−1.6 (0.1)	0.067
Listless speech (16)	2.1 (0.8)	−0.9 (0.1)	2.2 (0.9)	−1.0 (0.1)	0.857
Hypoactivity (17)	2.4 (1.1)	−1.2 (0.1)	2.4 (1.1)	−1.0 (0.1)	0.166

*CDRS-R* children’s depression rating scale-revised, *LS* least squares, *N* number of patients, *OFC* olanzapine/fluoxetine combination, *SD* standard deviation, *SE* standard error

## Discussion

Results from the present analysis indicated significant impairment in quality of life (QoL) among patients in an acute episode of pediatric bipolar depression. Although this may not be particularly surprising, it serves as an important reminder of the vulnerability of this population and the need for treatment during this phase of the illness, which may sometimes be overlooked relative to the more dramatic manic phase. The mean baseline KINDL-R domain scores in the pediatric population with bipolar depression reported here (KINDL-R patient-rated scale range 32.3 [self-esteem] to 55.5 [family]; KINDL-R parent-rated scale range: 29.9 [self-esteem] to 55.5 [physical well-being]) were very low in comparison to those of a normative school-aged reference population (range 66.6 [self-esteem] to 84.0 [family]) [32] but were also lower than those from another study evaluating a pediatric population diagnosed with bipolar disorder in any phase of the illness (range 44.9 [self-esteem] to 62.4 [emotional well-being]) [6]. The baseline scores were also much lower than those reported in a previous study with a population of children and adolescents with epilepsy from the United Kingdom (range 59.1 [friends] to 81.7 [family] [39]. This suggests that QoL in children/adolescents with bipolar depression is significantly impaired, a finding also supported by analyses of Freeman et al. [6]. Relative to the normative data (and according to the SDs from the normative data [6]), the population in the present study on average was at least 2 SDs worse than normal on their KINDL-R total score, emotional well-being and Friends subscale scores and was at least 1 or more SDs worse than normal on KINDL-R subscales of Self-esteem, Family, and School and on the Disease module.

With respect to treatment differences, although there were statistically significantly greater gains in quality of life in some QoL subscales for the OFC-treated patients relative to the placebo-treated patients, these differences were relatively modest in this 8-week study and varied somewhat by reporter (parent or child). Based on patients’ self-reports, there were greater improvements on the KINDL-R Self-esteem subscale score in the OFC-treated patients than the placebo-treated patients. Based on parents’ reports about their offspring, there were greater improvements on the KINDL-R subscale scores of Self-esteem, emotional Well-being, and Family. Across these few subscales, the difference in improvement between treatment groups was about 7 points. Although it is difficult to ascertain whether this difference between OFC and placebo is clinically significant, use of the rating anchors for the subscale items can provide some context for the findings. For instance, on the patient-rated Self-esteem subscale for the OFC-treated patients, the average scores were indicative of a change from feeling self-pride “never to seldom” at baseline to “seldom to sometimes” at endpoint, whereas the placebo-treated patients’ average results were indicative of a change from self-pride “never to seldom” at baseline to “seldom” at endpoint. Although subtle, this difference suggests clinically meaningful movement. However, because of the lack of adjustment for multiple comparison and as shown by the lack of statistical significance when controlling for CDRS-R total score, these findings were not statistically robust. Indeed, the effect sizes were small even in those subscales that showed significant improvement with treatment versus placebo. Nevertheless, visual comparison of the findings in this study relative to those of a normal sample (Fig. 1) also suggest that after 8 weeks of treatment, patients’ QoL scores were moving in the direction of “normal” but still below those of their healthy peers.

The KINDL-R questionnaire provides the ability to obtain a self-assessment in children and adolescents ranging from 8 to 12 years (Kid-KINDL), and 13 to 16 years (Kiddo-KINDL), and an external assessment of health-related QoL in children and adolescents ranging from 8 to 16 years (KINDL-R parent-rated). The parents are asked to complete the KINDL-R questionnaire with judgments from their own point of view of their children’s QoL. Because the children/adolescents and parents complete the questionnaires independently of one another, it is interesting to note the differences in perspectives. Patients tended to rate their QoL better at baseline than the parents did. However, parents almost universally rated better baseline to endpoint improvements than the patients, regardless of assigned treatment group. Previous studies also support the importance of obtaining the perspective of both the child and the parent when reporting on studies related to QoL in children [40, 41]. Limitations in insight observed in children may be the result of experiencing an acute depressive episode but may also be a function of the patients’ age and developing cognitive capabilities. In addition, it is possible that parents may be more prone to the placebo effect and would tend to report their belief that the blinded study medication is helping. Given the subjective nature of the KINDL-R, self-reporting by this young patient group, in particular for adolescents, might be a more valid approach to measuring QoL than a parent report. Previous studies have reported that adults (aged 45–85 years) with bipolar disorder are dissatisfied with their QoL even when they are in a state of remission, and in patients with bipolar disorder and schizophrenia in remission, there was a negative association between insight and physical domain [42]. In addition, in patients with depressive disorder, a high level of self-stigma was associated with poor QoL [43]. Including parent-reported measures in studies of bipolar disorder has been shown to add value to studies of treatment outcome by complementing clinician report measures and representing the parent’s perspectives and providing a more comprehensive picture of the child’s functioning [44]. Interestingly, a recent paper [45] that examined QoL using KINDL-R in 530 healthy children in Germany found that the perception of QoL has increased in both children and parent reports over the past 10 years. The authors noted that the largest increases occurred in self-esteem, physical well-being, and family, and speculated that this may be due to changes in the social and environmental life of the children. The study also found that QoL decreases with increasing age especially in girls, which may be attributed to increasing pressure in school and declining leisure time.

Significant improvements were observed in terms of severity of depression, as assessed by the CDRS-R. Item analysis of the CDRS-R indicated significantly greater improvement for the OFC group than the placebo group on 7 of 17 items on the CDRS-R, including 4 items that overlap conceptually with domains assessed by the KINDL-R. These 4 items were self-esteem, difficulty having fun, impaired schoolwork, and social withdrawal. This might suggest that much of the CDRS-R total score improvement was due to items that are more socially oriented. This conceptual overlap could also explain why none of the KINDL-R scores were significantly different between the OFC and placebo groups when the CDRS-R total (change from baseline) was included in the baseline adjustment of the KINDL-R analyses. The change in CDRS-R total score was placed into the KINDL-R statistical model as an explanatory variable. Although not shown, the analyses showed that the CDRS-R had a strong relationship with the KINDL-R results. Nevertheless, OFC improved depression in these pediatric patients with bipolar depression as noted not only by the significant improvement in item 11 (depressed feelings) of the CDRS-R, but also by the significant improvement in the bipolar depression rating scale and the clinical global impressions scale-bipolar version severity of depression as noted in the primary publication [15], although these 2 scales have not been validated in children and adolescents with bipolar depression. Of note, on the CDRS-R items (rated on a 0–5 scale) that were significantly improved for OFC compared to placebo, the between-group difference in mean change from baseline ranged from about 0.4–0.8 points. For example, for sleep disturbance the between-group difference in improvement was 0.8 points, with endpoint score being nearly 1 for OFC-treated patients (no difficulty or occasional difficulty sleeping) versus 2 at endpoint for placebo (frequent difficulty sleeping).

Few treatment options have proven to be effective for treating the depressive phase of bipolar disorder in children and adolescents. In addition to pharmacotherapy, it is important to consider the use of promising psychosocial interventions such as child- and family-focused cognitive behavioral therapy, dialectical behavioral therapy, interpersonal and social rhythm therapy, multifamily psychoeducation group psychotherapy, and family-focused treatment [46]. Psychosocial interventions have yielded positive results in combination with pharmacotherapy and may enhance or help maintain improvements in QoL. For example, West et al. [47] found that children and adolescents with bipolar disorder who were maintained on treatment and a child- and family-focused cognitive-behavioral therapy program for 3 years after the initial intervention showed significant long-term improvement in symptoms and psychosocial functioning relative to the control group that received medication and standard psychotherapy. Hlastala et al. [48] found that a dozen adolescents with bipolar disorder who received medication and also participated in 16–18 sessions of Interpersonal and Social Rhythm Therapy over a period of 20 weeks also showed substantial improvement in global functioning as well as on measures of psychiatric symptoms. Fifty-eight adolescents with either bipolar I, II, or not otherwise specified were assigned to either family focused therapy and pharmacotherapy or enhanced care and pharmacotherapy for up to 2 years [49]. Although recovery rates from the index episode and time to recurrence of depression were not different between groups, patients in the family focused therapy group showed faster recovery from baseline depressive symptoms, spent fewer weeks in depressive episodes and had a more favorable trajectory of depressive symptoms for 2 years.

The depressive phase in pediatric bipolar disorder is associated with various negative outcomes, such as suicidality, problem behaviors and hopelessness, and significant impairment in QoL [50], thus highlighting the urgency for needed intervention during the depressive phase. Changes from baseline to week 8 in the CDRS-R total score were significantly greater for OFC-treated compared with placebo-treated patients and significant between-group differences were also seen starting from week 1 and all subsequent visits up to week 8 [15]. Given the improvement in depressive symptoms [15] and improvement in some aspects of the QoL as shown with the KINDL-R, this suggests that OFC may be a treatment option for children and adolescents with bipolar depression; however, these results must be balanced against the safety results. Safety findings in the primary study were consistent with those observed in adults treated with OFC or adolescents treated with olanzapine, with the exception of a greater increase in QTc interval that was observed in this study [15]. Somnolence, weight gain, and increased appetite were the most common treatment-emergent adverse events reported in the OFC group, and weight gain was significantly greater for OFC- than placebo-treated patients [15].

There are potential limitations to the present analyses that need to be considered. Assessment of QoL was a secondary objective of the study [15], and findings were not adjusted for multiple comparisons. Therefore, the findings are not confirmatory and should be interpreted with caution. An active comparator was not used in this study so results should be interpreted accordingly. In addition, because the study did not include healthy controls, QoL scores have been shown relative to a pre-existing normative population assessed as part of the development and validation of the KINDL-R [32]. Although the mean ages for the 2 populations align well, differences in QoL scores between the populations could be due at least in part to cultural or geographic differences. Also, because the data for the healthy population were published over a decade before the results of the current pediatric bipolar depression study, the current analysis may be less reflective of the true differences between the present pediatric bipolar sample and a healthy pediatric sample today. Finally, the duration of this study was 8 weeks, which is relatively short when assessing QoL. It is unknown whether a longer study might have resulted in greater or lesser differences between drug and placebo on patient- or parent-rated QoL. Because there is a scarcity of QoL studies in children and adolescents with bipolar depression, it is difficult to put the present findings into context or compare outcomes.

## Conclusions

Pediatric patients with bipolar depression have a substantially reduced QoL. Although OFC-treated patients showed significant within-group improvement in QoL, differences versus placebo-treated patients were observed only in some aspects of QoL in the treatment of depressive episodes associated with bipolar I disorder in children and adolescents. However, after up to 8 weeks of treatment, patients’ QoL scores were still below the KINDL-R scores of a normal population. This highlights the need to consider QoL when treating patients with bipolar depression.

## Abbreviations

ANCOVA: analysis of covariance
CDRS-R: children’s depression rating scale-revised
DSM-IV-TR: Diagnostic and Statistical Manual of Mental Disorders, Fourth Edition, Text Revision
GCP: good clinical practice
KINDL-R: KINDL questionnaire for measuring health-related QoL in children and adolescents
Kid-KINDL: KINDL-R questionnaire self-assessment in children and adolescents age 8–12 years
Kiddo-KINDL: KINDL-R self-assessment in teens age 13–16 years
KINDL-R parent-rated: KINDL-R questionnaire external assessment of health-related QoL in children and adolescents 8–16 years
LS: least squares
MMRM: mixed-model repeated measures
OFC: olanzapine/fluoxetine combination
QoL: quality of life
SSRI: selective serotonin reuptake inhibitor
USFDA: US Food and Drug Administration
YMRS: Young Mania Rating Scale
